# Nrf2 and its signaling pathways in sepsis and its complications: A comprehensive review of research progress

**DOI:** 10.1097/MD.0000000000042132

**Published:** 2025-04-18

**Authors:** Huan Liu, Lei Wang, Jinhua Zhou

**Affiliations:** a Department of Emergency Internal Medicine, Jining NO.1 People’s Hospital, Jining, PR China; b Department of Pulmonary and Critical Care Medicine, Jining NO.1 People’s Hospital, Jining, PR China.

**Keywords:** inflammation, Nrf2, organ injury, oxidative stress, sepsis, treatment strategies

## Abstract

Sepsis is a life-threatening condition characterized by organ dysfunction resulting from a dysregulated host immune response to infection. It is associated with a high incidence, intricate pathophysiological mechanisms, and rapidly progressive severity, rendering it a leading cause of mortality among patients in intensive care units. The Nuclear Factor Erythroid 2-Related Factor 2 (Nrf2) is a transcription factor pivotal for maintaining cellular redox homeostasis by regulating the expression of antioxidant and cytoprotective genes. Emerging evidence suggests that activation of the Nrf2 signaling pathway attenuates sepsis-induced inflammatory responses, oxidative stress, and organ dysfunction, thereby improving clinical outcomes. These findings underscore the potential of Nrf2 as a therapeutic target, offering a promising avenue for the development of novel interventions aimed at mitigating the complications and improving the prognosis of sepsis.

## 1. Introduction

Sepsis is a leading cause of mortality among patients in intensive care units, driven by its high prevalence, intricate pathophysiology, and rapid clinical deterioration. It manifests as life-threatening organ dysfunction resulting from a dysregulated host immune response to infection, posing a significant global health challenge.^[1]^ Data from the 2017 Global Burden of Disease Study highlight the alarming impact of sepsis, with approximately 48.9 million cases and 11 million deaths annually, representing nearly 20% of all global deaths.^[2]^ The underlying mechanisms of sepsis are complex, encompassing dysregulated inflammatory responses, excessive oxidative stress, mitochondrial dysfunction, and apoptotic pathways, all of which contribute to progressive organ dysfunction and eventual failure.

Nuclear Factor Erythroid 2-Related Factor 2 (Nrf2) is a pivotal transcription factor that orchestrates the cellular defense system against oxidative stress and toxic insults. Through the activation of antioxidant response elements (AREs) and regulation of phase II detoxification enzymes, Nrf2 ensures the maintenance of redox homeostasis and supports cytoprotective functions under pathological conditions.^[3]^ Notably, preclinical studies have demonstrated that Nrf2 activation attenuates key drivers of sepsis-induced organ dysfunction, including hyperinflammatory responses^[4]^ and oxidative damage.^[5]^ These effects are particularly evident in mitigating complications such as acute lung injury, acute kidney injury, and sepsis-associated myocardial depression. Moreover, evidence suggests that Nrf2 activation enhances mitochondrial biogenesis, reduces cellular apoptosis, and promotes tissue regeneration, underscoring its integral role in maintaining organ integrity during sepsis..

## 2. Nrf2

NRF2, a member of the Cap’n’Collar subfamily of alkaline leucine zipper transcription factors, interacts with Kelch-like ECH-related protein 1 (Keap1) in a quiescent state, remaining sequestered in the cytoplasm.^[6]^ Upon exposure to oxidative stress or other stimuli, NRF2 dissociates from Keap1 and translocates into the nucleus. There, it binds to the promoters of genes involved in antioxidant stress, thereby facilitating their expression and bolstering the cell’s antioxidant defense mechanisms.^[7]^ Activation of the NRF2 pathway has been implicated in diverse physiological and pathological processes,including cancer,^[8]^ liver diseases,^[9]^ diabetes,^[10]^ and sepsis.^[11]^

**Figure 1.**
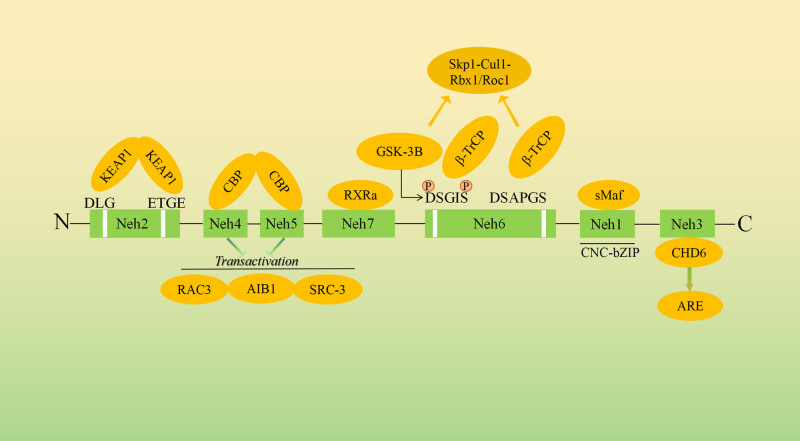
Nrf2 has 7 functional domains (Neh1–Neh7). Neh1 contains a bZIP structure for binding to sMaf, DNA, and transcription factors, and regulates Nrf2 stability via ubiquitin ligase. Neh2, with DLG and ETGE motifs, interacts with Keap1 to control Nrf2 ubiquitination. Neh3 binds to CHD6, regulating ARE gene expression. Neh4 and Neh5 interact with CBP and co-regulators (RAC3/AIB1/SRC-3) to modulate target genes, with Neh5 also sensing redox-sensitive nuclear export signals. Neh6, rich in serine, facilitates Keap1-independent degradation through interaction with β-transducin proteins and GSK-3β. Neh7 binds to RXRα, inhibiting the Nrf2-ARE pathway.

Nrf2 is a 66 kDa protein comprising 7 conserved functional regions known as Nrf2-ECH homology domains (Neh).^[12]^ Neh1 contains a basic leucine zipper structure, which allows Nrf2 to bind to small Maf (sMaf) proteins (K, G, and F), DNA, and other basic leucine zipper transcription factors. It plays a crucial role in recognizing AREs and activating gene transcription. Additionally, Neh1 can bind to ubiquitin ligase, regulating the stability and transcriptional activity of Nrf2.^[7]^ Meanwhile, Neh2, Located at the N-terminal, Neh2 contains DLG and ETGE motifs, both essential for interaction with Keap1. This interaction mediates Nrf2 ubiquitination and subsequent proteasomal degradation, serving as a key negative regulatory mechanism. The domain also contains several lysine residues that facilitate Nrf2 ubiquitination. Through these mechanisms, Neh2 controls Nrf2 stability and activity, linking Nrf2 to the cellular response to oxidative stress.^[13]^ The C-terminal Neh3 domain interacts with the transcriptional activator CHD6, thereby regulating the expression of ARE-related target genes. It works synergistically with Neh4 and Neh5, contributing to the transcriptional activation of Nrf2 target genes.^[14]^ Neh4 and Neh5 can bind to the transcriptional activator CBP and nuclear co-regulators such as RAC3/AIB1/SRC-3, playing a role in regulating Nrf2 target gene expression. Additionally, Neh5 can detect redox-sensitive nuclear export signals, controlling the intracellular localization of Nrf2 and influencing its activity in response to oxidative stress. Neh6 contains DSGIS and DSAPGS motifs, which serve as phosphorylation targets for glycogen synthase kinase-3β. This phosphorylation event facilitates the formation of a ubiquitin ligase complex with β-transducin repeat-containing proteins and Skp1-Cul1-Rbx1/Roc1, creating a Keap1-independent pathway for Nrf2 ubiquitination and degradation. This pathway provides an additional regulatory mechanism independent of Keap1.^[15]^ Additionally, the Neh7 interacts with the nuclear receptor retinoid X receptor α, leading to the inhibition of the Nrf2-ARE signaling pathway. This interaction adds another layer of control over Nrf2 activity, linking it to broader cellular signaling networks^[16]^ (Fig. 1).

The regulatory mechanism of Nrf2 encompasses multiple levels of regulatory factors and target genes. Nrf2 activation primarily occurs in response to oxidative stress or chemical stimuli. Keap1, acting as a significant negative regulator within the cell, maintains Nrf2 in an inactive state and facilitates its degradation via the ubiquitination degradation pathway. Upon exposure to oxidative stress or similar triggers, the interaction between Keap1 and Nrf2 is disrupted, enabling Nrf2 to detach from Keap1 and translocate into the nucleus, thereby initiating the antioxidant stress response.^[17]^ Once activated, Nrf2 binds to the promoters of antioxidant stress genes within the nucleus, thereby promoting their transcription and expression. These target genes encompass antioxidant enzymes^[18,19]^ (e.g., superoxide dismutase and catalase), detoxification enzymes^[20]^ (e.g., glutathione-S-transferase), and metabolic enzymes^[21]^ (e.g., NAD(P)H: quinine reductase), whose expression enhances cellular resistance to oxidative stress and preserves cellular redox balance. Overall, the regulatory mechanism of Nrf2 involves activating the Keap1/Nrf2 signaling pathway and regulating the transcription of antioxidant stress genes by Nrf2, playing a pivotal role in cellular protection against oxidative stress and the maintenance of cellular homeostasis.

Regulation of the Nrf2 signaling pathway predominantly revolves around Nrf2 activation and the alleviation of Keap1 inhibition. Oxidative stress stands out as a chief activator of the Nrf2 signaling pathway. Upon oxidative stress induction in the cell, Nrf2 is liberated and shuttles into the nucleus, facilitating the transcription of antioxidant response genes.^[22]^ Furthermore, inflammatory responses and tissue injuries can also prompt the activation of the Nrf2 signaling pathway.^[23]^ Activation of the Nrf2 signaling pathway additionally entails the alleviation of Keap1 inhibition. Keap1, functioning as a negative regulator of Nrf2, curtails its activity through binding and subsequent degradation.^[24]^ Nevertheless, certain compounds and substances can covalently bind to Keap1, diminishing its affinity for Nrf2, thereby freeing Nrf2 and enhancing its activity.

Beyond its role in antioxidant response, the Nrf2 signaling pathway also modulates cellular anti-inflammatory responses and promotes survival. Nrf2 exerts its anti-inflammatory effects by modulating the expression of an array of inflammatory factors.^[25]^ Furthermore, Nrf2 is intricately involved in regulating cell survival and apoptosis.^[26]^ Research has evidenced that activation of the Nrf2 signaling pathway can bolster cell survival rates and confer protection against various diseases such as pneumonia,^[27]^ myocardial infarction,^[28]^ and stroke.^[29]^ In summary, the Nrf2 signaling pathway intricately regulates cellular self-protective mechanisms by modulating antioxidant response, anti-inflammatory responses, and cell survival. Activation of this pathway holds promise in enhancing cellular resilience to oxidative stress, inflammation, and injury, thereby potentially offering therapeutic benefits for a spectrum of diseases.

## 3. Nrf2 and sepsis

Lipopolysaccharides (LPS) constitute a distinctive element of the cell wall in Gram-negative bacteria, commonly referred to as endotoxins, with the capacity to induce sepsis, septic shock, and multiple organ dysfunction syndrome.^[30]^ LPS exerts its inhibitory effect on glutathione synthesis through the attenuation of protein ubiquitination modification involving Nrf2 and MafG, consequently heightening susceptibility to reactive oxygen species (ROS)-mediated damage and precipitating organ dysfunction.^[31]^ Research findings indicate that the upregulation of ubiquitin ligase 9, pivotal for Nrf2 protein ubiquitination, or direct activation of Nrf2 by antioxidant compounds such as dihydroquercetin, can counteract Nrf2 inhibition, thereby conferring protective benefits and enhancing survival rates in murine models of inflammation.^[32]^ Moreover, induction of the Nrf2 target gene heme oxygenase-1 (HO-1) has demonstrated a protective role in murine endotoxemia.^[33]^ Comparative analysis reveals heightened inflammation and a worsened prognosis in mice with Nrf2 gene knockout, contrasting with the ameliorated inflammation and enhanced survival rates observed in mice treated with cecal ligation and puncture following the reduction of endogenous Nrf2 levels mediated by the physiological regulator Keap1.^[34]^ Additionally, in the cecal ligation and puncture model, administration of antioxidants, including resveratrol,^[35]^ mangiferin,^[36]^ myricetin,^[37]^ dimethyl fumarate^[38]^ and ascorbic acid,^[39]^ to facilitate Nrf2 activation, manifests a reduction in organ damage and an improvement in survival rates. Beyond its protective effects against tissue damage instigated by oxidative stress and inflammation, Nrf2 activation augments the antibacterial capacity of macrophages, thereby exerting a protective influence on the onset and progression of sepsis.^[40,41]^ In conclusion, the Nrf2 pathway assumes a pivotal role in mitigating inflammation, oxidative stress, and organ damage attendant upon sepsis. The modulation of Nrf2 activity through diverse compounds or therapeutic modalities holds potential for enhancing the prognosis of sepsis patients.

## 4. Nrf2 and sepsis related complications

### 4.1. Sepsis-associated acute lung injury

Acute lung injury (ALI) is a severe respiratory disease characterized by diffuse, non-cardiogenic pulmonary edema resulting from alveolar injury. It presents clinically with refractory hypoxemia and respiratory distress.^[42]^ The pathogenesis of ALI involves oxidative stress and an inflammatory response. Disruption of the integrity of the alveolar capillary barrier or activation of the inflammatory response can lead to lung inflammation and injury.^[43]^ Despite decades of research on ALI/ARDS, it remains a life-threatening clinical syndrome worldwide due to the lack of effective treatment strategies.^[44]^ ALI can be caused by multiple pathogenic factors through direct or indirect damage to the inflammatory response. Among these factors, sepsis is the main risk factor for ALI, with inflammatory and endothelial dysfunction being characteristic features of both sepsis and ALI/ARDS.^[45]^

Nrf2 is a transcription factor known for protecting the lungs from oxidative stress. Upon stimulation by oxidative stress, Nrf2 migrates from the cytoplasm to the nucleus and binds to the antioxidant stress response element, initiating the transcription of antioxidant genes, including glutathione peroxidase, superoxide dismutase (SOD), to eliminate free radicals and alleviate oxidative stress damage to lung tissue.^[20]^ In terms of inflammation regulation, Nrf2 regulates the expression of inflammation-related genes, such as cytokines like interleukin-6 (IL-6) and tumor necrosis factor-alpha (TNF-α), and inflammatory mediators like interleukin-1 beta (IL-1β), to inhibit the development of inflammatory reactions by inducing inflammation, thereby alleviating inflammation damage and worsening of lung tissue.^[46]^ Specific single nucleotide polymorphisms within the NRF2 gene can affect the activity and binding affinity of its promoter, which is associated with an increased risk of ALI.^[47]^

Multiple studies have explored the role of the Nrf2 signaling pathway in sepsis-associated ALI and its potential therapeutic significance. Lai et al^[48]^ showed that uridine inhibited ferroptosis of macrophages by activating the Nrf2 signaling pathway, alleviating lung injury, inhibiting inflammation and lipid peroxidation, and improving sepsis-induced ALI. During the inflammatory response process, the NRF2 pathway can be activated by multiple signaling pathways. The PI3K/AKT pathway is an important upstream regulatory factor of NRF-2/HO-1.^[49]^ Songorine reduces cell apoptosis in lung tissue of sepsis mice induced by lipopolysaccharide through the PI3K/AKT/NRF2 signaling pathway, lowers the levels of pro-inflammatory cytokines, and exerts anti-apoptotic, anti-inflammatory, and antioxidant effects, thereby alleviating ALI induced by sepsis in mice.^[50]^ Sirtuin1 (SIRT1), as a cellular nicotinamide adenine dinucleotide (NAD+)-dependent deacetylase, plays a variety of roles in cell biological processes, such as DNA repair, aging, apoptosis, inflammation, and cell metabolism.^[51,52]^ Echinoside can activate SIRT1 and promote its expression. The activated SIRT1 competitively binds to p22Pox, inhibiting the activation of NOX4 and promoting its ubiquitination and degradation.^[53]^ In addition, SIRT1 deacetylates Nrf2, promotes downstream expression of antioxidant enzymes, alleviates oxidative stress-induced endothelial cell pathological activation and mitochondrial pathway apoptosis, thereby inhibiting acute lung injury and oxidative stress caused by sepsis.^[54]^ Overall, research on Nrf2 signaling in sepsis-associated ALI continues to uncover novel mechanisms and potential therapeutic targets for mitigating oxidative stress, inflammation, and injury in the lungs.

### 4.2. Sepsis-induced myocardial injury

Myocardial injury represents the predominant and gravest complication of sepsis, affecting over 50% of sepsis patients with varying degrees of cardiac dysfunction in early stages, correlating with mortality rates of 70% to 90%.^[55]^ However, the precise mechanisms underlying myocardial injury in sepsis remain incompletely understood. Sepsis induces oxidative stress in patients, potentially resulting in myocardial damage, a phenomenon extensively documented in literature.^[56]^

Nrf2, a pivotal factor in antioxidant stress signaling pathways, emerges as a key player in this process. During sepsis-induced myocardial injury, Nrf2 expression is suppressed, impeding the activation of the CTRP1 binding site and facilitating cardiomyocyte pyroptosis due to diminished protection against oxidative damage.^[57]^ In lipopolysaccharide-stimulated cardiomyocytes, ROS levels rise concomitantly with decreased Nrf2 expression, whereas overexpression of CTRP12 in cardiomyocytes enhances Nrf2 expression, mitigating ROS levels, inhibiting pro-inflammatory cytokine transcription and release, and alleviating lipopolysaccharide-induced cardiomyocyte damage.^[58]^ The excessive release of reactive oxygen species and inflammatory factors emerges as the primary pathogenic mechanism underlying sepsis-induced myocardial injury. S1R exhibits potential in clearing ROS,^[59]^ reducing mitochondrial oxidative stress^[60]^ and myocardial apoptosis,^[61]^ thus alleviating sepsis-induced myocardial injury via the Nrf2/HO1 signaling pathway.^[62]^ Moreover, elevated IL-16 expression observed in myocardial cells and serum of septic mice compared to non-septic mice can be modulated by anti-IL-16 neutralizing antibodies through the Nrf2 pathway, thereby reducing myocardial cell apoptosis, enhancing cardiac function, and lowering septic mortality rates.^[63]^

Myocardial injury resulting from sepsis predisposes individuals to cardiac dysfunction and malignant arrhythmias. Astilbin and Crocetin, both possessing anti-inflammatory and cytotoxic effects, offer potential therapeutic interventions. Astilbin activates the NRF2/HO-1 pathway, inhibiting the TLR4/NF-κ B pathway, reducing myocardial electrical remodeling, and promoting the expression of gap junction protein and ion channels, thereby decreasing susceptibility to ventricular fibrillation.^[64]^ Crocetin, a natural compound, exhibits anti-inflammatory and cytotoxic properties in the context of myocardial ischemia-reperfusion injury.^[65]^ It mitigates the levels of tumor necrosis factor-α, IL-1, IL-6, and IL-8 mRNA by modulating the p65/Keap1 signaling pathway and activating the Nrf2/HO-1/NQO1 signaling pathway. Moreover, Crocetin demonstrates protective effects against LPS-induced mitochondrial respiration, free fatty acid β oxidation, and mitochondrial morphology alterations in H9c2 cells. In conclusion, Crocetin ameliorates endotoxin-induced cardiac dysfunction through its dual regulatory role in inflammatory responses and mitochondrial function.^[66]^

### 4.3. Sepsis-related liver injury

The liver plays a vital role in maintaining immune and metabolic balance during sepsis.^[67]^ Sepsis-related liver injury encompasses liver damage resulting from sepsis, manifesting in abnormal biochemical markers, liver dysfunction, and potential liver failure.^[68]^ The sustained inflammation and oxidative stress in sepsis, driven by cytokine release, neutrophil activation, and oxygen free radical production, contribute to liver damage.^[69]^ LPS induces inflammation during sepsis by inhibiting the expression of Nrf2 and heme oxygenase-1 (HO-1), while antioxidants such as ATT activate the Nrf2/GPX4 and NF-κB pathways, reducing LPS-induced liver injury and oxidative stress.^[70]^ Similarly, In sepsis-induced liver injury, 6-gingerol mitigates the inhibition of Nrf2 and HO-1 expression caused by LPS and ATP stimulation through activation of the Nrf2 signaling pathway. This activation promotes IL-1β secretion and reduces caspase-1, consequently lowering levels of biochemical markers ALT and AST, mitigating liver tissue damage and cell death, diminishing inflammatory response, and inhibiting oxidative stress response.^[71]^

Astragaloside IV, derived from the traditional Chinese herb Astragalus membranaceus, has gained attention in biomedical research due to its varied pharmacological properties, including anti-inflammatory, antioxidant, immunomodulatory, and hepatoprotective effects.^[72]^ In Sepsis-related liver injury, AS-IV inhibits lipopolysaccharide-induced NLRP3 inflammasomes and reverses LPS-induced pro-inflammatory cytokine IL-1β by activating the SIRT1/Nrf2 pathway and decreasing TNF-α and IL-6 expression. This alleviates pathological changes in liver tissue, decreases serum levels of ALT and AST, inhibits cell apoptosis in liver tissue, and mitigates lipopolysaccharide-induced oxidative stress and inflammatory response.^[73]^ Other research results indicate that Dexmedetomidine activates α2AR, inhibiting glycogen synthase kinase-3β activity, thereby regulating the MKP-1/Nrf2 signaling pathway, enhancing antioxidant capacity, reducing ALT, AST, and T-BIL levels, and mitigating lipopolysaccharide-induced liver oxidative stress and cell apoptosis in rats.^[74]^ Additionally, PPARγ activation of Nrf2 attenuates ROS damage, inhibits the expression of the TXNIP/NLRP3 signaling pathway, reduces liver cell death mediated by caramel isomerase due to abdominal infection, and alleviates sepsis-induced liver injury.^[75]^ MaR1, derived from ω-3 polyunsaturated fatty acids, inhibits neutrophil infiltration, promotes macrophage phagocytosis of dead cells,^[76]^ enhances tissue regeneration during acute inflammation, inhibits the mitogen-activated protein kinase/NF-κB signaling pathway and NLRP3 inflammasome-induced pyroptosis, activates macrophage M1/M2 polarization, and activates the Nrf2/HO-1 signaling pathway, thereby mitigating LPS/D-GalN-induced ALI inflammation.^[77]^ In summary, Nrf2 activation protects the liver from oxidative stress and inflammation by enhancing intracellular antioxidant and detoxification abilities and inhibiting inflammatory responses.

### 4.4. Sepsis-associated acute kidney injury

Sepsis-related acute kidney injury (S-AKI) is a severe complication among critically ill patients with sepsis, with over 60% of sepsis or septic shock patients developing S-AKI.^[78]^ Its mechanism involves microcirculatory disturbances, inflammation, oxidative stress, and damage to renal tubular epithelial cells.^[79,80]^ Research indicates that acute kidney injury induced by septic shock in rats is regulated through the PI3K/Nrf2 pathway. Inhibition of the PI3K/Nrf2 pathway may exacerbate acute kidney injury caused by septic shock, while HO-1, as an effector protein activating PI3K/Nrf2, plays a protective role in regulating AKI induced by septic shock in rats.^[81]^ Mitochondrial damage and ROS also play significant roles in the pathogenesis of sepsis-related acute kidney injury.^[82]^ Mitochondrial damage and ROS also play significant roles in the pathogenesis of sepsis-related acute kidney injury.^[83]^ Mitochondrial damage compromises the main function of mitochondria in renal tubular epithelial cells, leading to the release of ROS within the cells, thereby causing damage. The antioxidant procyanidin B2 is associated with the improvement of mitochondrial dynamics and increased nuclear translocation of the transcription factor Nrf2. Treatment with procyanidin B2 can enhance the expression of the second phase detoxifying enzymes HO1 and NQO1 in the kidney, improve mitochondrial function, reduce mitochondrial damage by increasing the nuclear translocation of Nrf2, promote mitochondrial biogenesis, and improve mitochondrial dynamics, thereby reducing mitochondrial damage and related cell apoptosis, thus exerting a protective effect on acute kidney injury.^[84]^ LPS-mediated inflammation and oxidative stress partially contribute to renal injury in S-AKI.^[85]^ LPS, present in the outer membrane of Gram-negative bacteria, triggers inflammation mediated by the Toll-like receptor 4 (TLR4) complex in immune cells, endothelial cells, and tubular cells of the kidneys, releasing cytokines such as IL-1, TNF-α, and IL-6.^[86,87]^ Mitophagy, the selective degradation of damaged mitochondria under mitochondrial toxicity conditions, plays a vital role in renal injury in S-AKI. 4-OI can alleviate inflammation and oxidative stress and enhance mitophagy by activating the Nrf2 signaling pathway and inhibiting the STAT3 signaling pathway, thereby exerting its protective effect on the kidneys.^[85]^ Nrf2, as an essential intracellular antioxidant stress factor, is activated in S-AKI. Its primary function is to protect cells from oxidative damage by regulating the balance of oxidation and reduction. Under normal conditions, Nrf2 is negatively regulated by Keap1 and remains at a low level. However, under conditions such as oxidative stress, the activity of Keap1 is inhibited, leading to the release and activation of Nrf2, thereby initiating the antioxidant stress response. Nrf2 binds to the promoter region of AREs, regulating the expression of a series of antioxidant stress and cell protection genes, including oxidoreductases and heat shock proteins, thereby enhancing the cell’s antioxidant capacity.^[88]^ Its activation can promote the expression of antioxidant enzymes, clear free radicals, reduce oxidative damage, inhibit the production of inflammatory factors, alleviate inflammatory reactions, and promote cell repair and regeneration, thereby protecting renal function.^[89]^ In conclusion, the Nrf2 signaling pathway significantly contributes to kidney injury associated with sepsis. Firstly, Nrf2 shields the kidneys from oxidative stress by scavenging free radicals and mitigating oxidative harm. Secondly, it suppresses the generation of inflammatory mediators, thereby alleviating the renal damage induced by inflammatory responses. Moreover, Nrf2 modulates the expression of antioxidant and detoxification enzymes, bolstering the kidney’s capacity to counteract oxidative stress and metabolize toxic compounds. Consequently, activation of the Nrf2 signaling pathway presents a promising approach for preventing and managing sepsis-induced kidney injury, offering novel therapeutic avenues.

### 4.5. Sepsis-associated encephalopathy

Sepsis-associated encephalopathy (SAE) is a severe neurological complication commonly observed in sepsis patients, yet its pathophysiological mechanisms remain incompletely understood. The occurrence of SAE is intricately linked to various factors, including the release of inflammatory mediators, dysregulation of cerebral vascular autoregulation, impairment of blood-brain barrier function, and neuronal apoptosis.^[90]^ During sepsis, the body generates a significant amount of inflammatory mediators, such as TNF-α,^[91]^ IL-1β,^[92]^ and in IL-6,^[93]^ which can directly penetrate the blood-brain barrier, thereby affecting brain tissue and causing abnormal brain function. Moreover, damage to vascular endothelial cells and dysregulation of cerebral vascular autoregulation represent significant pathogenic mechanisms of SAE, leading to inadequate cerebral blood flow and microcirculatory disturbances, ultimately exacerbating brain tissue damage.^[94]^ Additionally, impairment of the blood-brain barrier results in the infiltration of inflammatory cells and release of inflammatory mediators in brain tissue, further exacerbating the pathological process of SAE.^[95]^ Oxidative stress and inflammation emerge as primary contributors to sepsis-induced cognitive impairment.^[96]^ Geniposide, by modulating the Nrf2 signaling pathway, upregulates the expression of HO-1 and NQO-1 genes. This downregulates the levels of malondialdehyde and oxidative proteins while augmenting the activity of SOD and catalase. Moreover, it attenuates hippocampal concentrations of TNF-α and IL-1β, myeloperoxidase activity, and NF-kB protein levels in septic rats. This suppression of oxidative stress and inflammatory reactions markedly ameliorates cognitive dysfunction in septic rats, thereby enhancing survival rates.^[97]^

Inflammation plays a pivotal role in disrupting the blood-brain barrier and constitutes a fundamental mechanism in the pathogenesis and progression of SAE.^[96]^ Inflammatory cytokines penetrating the brain further stimulate microglial cells, triggering a heightened cascade of inflammatory responses. This cycle of inflammation and activation of microglial cells results in significant neuronal damage. GYY4137 activates the Nrf2/ARE pathway by sulfhydrylating Keap1, thereby augmenting the expression of downstream antioxidant enzymes such as HO-1 and NQO1. Consequently, it impedes the degradation of tight junction proteins in the blood-brain barrier, thus diminishing levels of inflammatory factors in both serum and brain tissue. Moreover, it suppresses the activation of microglial cells and the release of inflammatory mediators, thereby reducing the expression levels of neuronal damage markers, thereby safeguarding the integrity of the blood-brain barrier and ameliorating the clinical outcomes of sepsis.^[98]^ Cellular apoptosis also contributes to the pathogenesis of SAE. Hydrogen gas enhances the expression of NRF2 and inhibits the expression of NLRP3, caspase-1, as well as cytokines IL-1β and IL-18 induced by SAE, thereby mitigating inflammation, neuronal apoptosis, and mitochondrial dysfunction in SAE tissues, consequently enhancing cognitive function.^[99]^ Additionally, hydrogen can augment the binding between Nrf2 and YY1, facilitate HO-1 expression, elevate levels of mitochondrial homeostasis-related proteins, safeguard glial cells, and alleviate sepsis-related encephalopathy.^[100]^ Fundamental research suggests that in SAE rats, the levels of oxidative stress and lipid peroxidation in hippocampal neurons escalate, accompanied by an increase in neuronal iron death, ultimately resulting in decreased cognitive function.^[101]^ Inhibition of iron death diminishes oxidative damage and fosters the activation of the Nrf2/HO-1 signaling pathway, consequently decreasing the expression levels of TNF-α and IL-1β, alleviating cognitive dysfunction, neuronal functional deficits, damage to blood-brain barrier integrity, and neuroinflammation, thereby conferring protection against the effects of SAE.^[102]^

### 4.6. Sepsis-related intestinal dysfunction

During sepsis, LPS triggers an upsurge in intestinal cell apoptosis, compromising the integrity of the intestinal mucosal barrier.^[103]^ This breach permits submucosal components to interface with the external milieu, facilitating the ingress of bacteria, toxins, and other pathogens through the intestinal wall, leading to tissue penetration and inflammatory reactions. Furthermore, excessive apoptosis of endothelial cells disrupts the integrity of the vascular endothelium, augmenting vascular permeability and precipitating fluid and cell extravasation, thereby exacerbating tissue edema and inflammatory responses.^[104]^ Berberine attenuates the formation of NLRP3 inflammasomes, inhibits NF-κB and PI3K/AKT signaling pathways, suppresses the release of inflammatory mediators, bolsters intracellular antioxidant capacity, thereby mitigating oxidative stress and inflammatory responses, fostering the restoration of the intestinal barrier and reinstating normal function of intestinal epithelial cells, and shielding cells from damage. Additionally, berberine curtails LPS-induced oxidative stress and ROS accumulation, manifesting antioxidant effects by modulating the Nrf2/Keap1 signaling pathway. Moreover, palmatine curbs LPS-induced autophagy of intestinal cells by inhibiting the PI3K/Akt/mTOR signaling pathway, hence diminishing excessive intestinal cell apoptosis.^[105]^ VDR serves as a pivotal regulatory factor for intestinal cell proliferation, barrier function, and immunity, while the VDR/Nrf2/HO-1 signaling pathway assumes a pivotal role in sepsis-related intestinal mucosal damage. Emodin heightens the expression levels of VDR and downstream pathways Nrf2 and HO-1 through the VDR/Nrf2/HO-1 signaling pathway. It suppresses the expression of TNF-α, IL-6, and malondialdehyde in serum and tissues, and augments the levels of SOD and GSH to counteract sepsis-related damage induced by abdominal puncture-induced sepsis, thereby preserving intestinal mucosal barrier function. Emodin treatment substantially boosts the mRNA and protein levels of ZO-1, Occludin, and Claudin-1, ameliorating intestinal mucosal molecular expression, and mitigates intestinal barrier dysfunction by upregulating the level of TJ proteins in intestinal tissues.^[106]^

## 5. Future prospects and therapeutic potential

The treatment of sepsis remains challenging due to the lack of targeted therapies, with current approaches relying mainly on antibiotics and organ support. The Nrf2 signaling pathway, which regulates oxidative stress and inflammation, is a promising therapeutic target. Its activation enhances antioxidant defenses and reduces cytokine release, potentially mitigating organ damage caused by sepsis. Synergistic effects may exist between Nrf2 activation and current adjunctive therapies. For example, blood purification techniques, such as continuous renal replacement therapy, not only remove inflammatory mediators but may also indirectly activate Nrf2 by reducing oxidative stress. Similarly, recombinant human alkaline phosphatase and CER-001 high-density lipoprotein infusions have demonstrated anti-inflammatory and antioxidant effects, which could complement Nrf2 activation.^[107–109]^

## 6. Conclusion and perspectives

Sepsis, characterized by life-threatening organ dysfunction stemming from an imbalanced host response to infection, remains a major cause of mortality among intensive care unit patients. The intricate pathogenesis and swift progression of sepsis necessitate novel therapeutic strategies to improve patient outcomes. Nrf2 emerges as a crucial player in mitigating sepsis-induced inflammatory damage, oxidative stress, and organ injury. Activation of the Nrf2 pathway presents promising avenues for therapeutic intervention, not only in sepsis but also in its related complications. Studies elucidate the protective effects of Nrf2 activation in conditions such as sepsis-associated acute lung injury, sepsis-induced myocardial injury, sepsis-related liver injury, sepsis-associated acute kidney injury, and sepsis-associated encephalopathy. Through its regulation of antioxidant response genes and modulation of inflammatory responses, Nrf2 activation holds significant potential in attenuating tissue damage and improving patient survival in sepsis and its associated complications. The exploration of Nrf2 signaling pathways offers valuable insights into the development of targeted therapies aimed at mitigating the detrimental effects of sepsis, thereby enhancing patient outcomes and reducing mortality rates. Further research into Nrf2-targeted interventions may pave the way for innovative treatment strategies in the management of sepsis and its related complications.

## Author contributions

**Conceptualization:** Huan Liu.

**Investigation:** Lei Wang.

**Methodology:** Lei Wang, Jinhua Zhou.

**Supervision:** Jinhua Zhou.

**Writing – original draft:** Huan Liu.

**Writing – review & editing:** Jinhua Zhou.
